# Comparative Effects of *Glycine max* and *Glycine soja* Leaves on *Clanis bilineata tsingtauica* Rearing Performance

**DOI:** 10.3390/ijms27083442

**Published:** 2026-04-11

**Authors:** Ping Zhao, Chen Meng, Syeda Wajeeha Gillani, Xueli Lu, Xi Jia, Meng Wang, Yu Bai, Yiru Song, Hongyan Hou, Yiqiang Li, Lu Wang, Zongchang Xu

**Affiliations:** 1 Jilin Provincial Key Laboratory of Plant Resource Science and Green Production, Jilin Normal University, Siping 136000, China; zp0713@jinu.edu.cn; 2 Qingdao Key Laboratory of Coastal Saline-Alkali Land Resources Mining and Biological Breeding, National Center of Technology Innovation for Comprehensive Utilization of Saline-Alkali Land, Marine Agriculture Research Center, Tobacco Research Institute of Chinese Academy of Agricultural Sciences, Qingdao 266100, China; mengchen01@caas.cn (C.M.);; 3 Yellow River Delta Modern Agriculture Research Institute, Shandong Academy of Agricultural Sciences, Dongying 257091, China; 4 College of Agronomy, Qingdao Agricultural University, Qingdao 266109, China

**Keywords:** wild soybean, nutritional ingredient, intestinal microflora, phyllosphere microbiota, edible insect

## Abstract

In China, the substantial gap between domestic soybean supply and growing consumption necessitates large-scale soybean imports. The use of cultivated soybean (*Glycine max*) leaves as feed for the edible insect *Clanis bilineata tsingtauica* reduces crop yield, posing a threat to national soybean production security. To address this issue, this study evaluated wild soybean (*Glycine soja*) as a potential alternative feed source. Comparative analyses examined the nutritional and anti-nutritional properties of *G. max* (cv. Qihuang34) and a laboratory-preserved *G. soja* germplasm, together with their effects on larval growth performance, nutritional composition, and associated microbiota. *G. soja* leaves exhibited significantly higher crude fat (5.61% vs. 2.17%), ash (11.07% vs. 9.62%), neutral detergent fiber (23.75% vs. 21.00%), calcium (4.05 g/kg vs. 3.41 g/kg), and phosphorus (2.52 g/kg vs. 2.38 g/kg) than *G. max* leaves, along with lower trypsin inhibitor levels (*p* < 0.01) despite higher phytic acid content (*p* < 0.05). Fifth-instar larvae reared on *G. soja* leaves showed a 12.9% greater body weight (6.846 g vs. 6.066 g), higher crude protein (672.14 g/kg vs. 555.02 g/kg), total soluble sugar (21.27 mg/g vs. 8.96 mg/g), and soluble protein (26.35 mg/g vs. 24.71 mg/g), but lower crude fat (187.44 g/kg vs. 205.82 g/kg, *p* < 0.05). 16S rRNA sequencing revealed distinct phyllosphere microbial communities, with *G. soja* enriched in diverse taxa (e.g., *Bacteroidota*, *Proteobacteria*) and *G. max* dominated by *Firmicutes*. Corresponding differences were observed in larval gut microbiota, and positive correlations suggested potential microbial transfer from *G. soja* leaves to larval guts. Overall, *G. soja* represents a promising alternative feed source for *C. bilineata*, reducing competition with soybean grain production and supporting sustainable insect farming.

## 1. Introduction

Rapid population growth has intensified global demand for sustainable food sources to ensure long-term food security [1]. Edible insects have attracted increasing attention due to their short rearing cycle, efficient food conversion, and high protein content [2]. As a result, they are considered a promising alternative animal protein source with the potential to help meet rising global protein demand [3,4]. Consistent with this trend, the global edible insect market continues to expand [5].

*Clanis bilineata tsingtauica*, a member of the family Sphingidae within the order Lepidoptera, is an edible insect widely consumed in China [6,7]. The larvae, which feed on nutrient-rich soybean (*Glycine max*) leaves, are valued for their high protein content and levels of unsaturated fatty acids (UFAs) and vitamins [8,9], nutrients associated with brain development, protection against cellular degeneration, and endocrine regulation in humans. However, stable industrial production remains constrained by challenges such as pest outbreaks, disease incidence, and photoperiod sensitivity, hindering a constant supply.

Feeding behavior in phytophagous insects is strongly influenced by the nutritional composition of host plants [10]. Host nutrient profiles determine insect growth, development, and ultimately their nutritional quality. Numerous species exhibit nutrition-driven host selection, such as the western flower thrips (*Frankliniella occidentalis*) shifting to nutrition-rich pollen [11] and *Bradysia* spp. preferring protein-rich chives (*Allium tuberosum*) and broad beans (*Vicia faba*) [12]. Nitrogen-rich plants are also favored by species such as gypsy moth (*Lymantria dispar*) [13,14].

Microbial communities associated with the phyllosphere and insect guts are important for understanding nutrient metabolism, immune regulation, and host-microbe interactions [15,16]. The phyllosphere harbors diverse microbial taxa that influence plant health and nutrient composition [17,18]. Once food enters the insect body, gut-colonizing microorganisms are closely linked to nutrient assimilation and physiological processes [19]. Gut bacteria have been shown to influence insects’ host-plant preference, assisting them in identifying suitable plants for survival and reproduction [20]. Furthermore, the feeding characteristics of insects can significantly alter the structure of their gut microbiota, which in turn affects their growth and development [21]. Recent microbiological studies highlight the pivotal role of diet and environmental factors in the assembly of the insect gut microbiota [22,23].

Wild soybean (*Glycine soja*), the progenitor of cultivated soybean (*Glycine max*) [24], is characterized by strong stress resistance, high adaptability, and a wide geographic distribution [25,26]. Its leaves contain higher concentrations of major free sugars, including glucose and sucrose, than those of cultivated soybean [27]. Despite this potential, no study has systematically evaluated *G. soja* leaves as a feed source for *C. bilineata* rearing. To evaluate the potential of *G. soja* as an alternative feed source for *C. bilineata*, this study compared the nutritional and anti-nutritional profiles of both soybean types and assessed their effects on *C. bilineata* larval performance and gut microbiota. Differences in phyllosphere microbial communities between the two soybean species were also examined, providing a foundation for microbial management strategies aimed at enhancing *C. bilineata* production. Therefore, evaluating alternative host plants that do not compete with grain production is essential for both food security and the sustainable development of the edible insect industry. By demonstrating that *G. soja* can effectively support larval growth and nutritional quality, this study provides a basis for reducing pressure on cultivated soybean resources, thereby contributing to global food security.

## 2. Results

### 2.1. Leaf Nutritional Components of Glycine soja and Glycine max

Leaf nutritional composition is a key indicator of forage quality. To compare wild soybean (*G. soja*) and cultivated soybean (*G. max*), we quantified the nutritional composition of their leaves. As summarized in Table 1, *G. soja* leaves exhibited a crude fat content of 5.61%, approximately 2.58-fold higher than that of *G. max* leaves (2.17%; *p* < 0.01). Ash, neutral detergent fiber, and calcium contents were also significantly higher in *G. soja* leaves (*p* < 0.01). Phosphorus content was also significantly higher in *G. soja* leaves than in *G. max* leaves (*p* < 0.05). Conversely, crude fiber content was significantly higher in *G. max* leaves (*p* < 0.01). No significant differences were observed in crude protein or acid detergent fiber between the two soybean types. Overall, these findings indicated that *G. soja* leaves exhibit higher values for several key nutritional indicators than *G. max* leaves.

### 2.2. Main Anti-Nutritional Components in Leaves of Glycine soja and Glycine max

**Figure 1:**
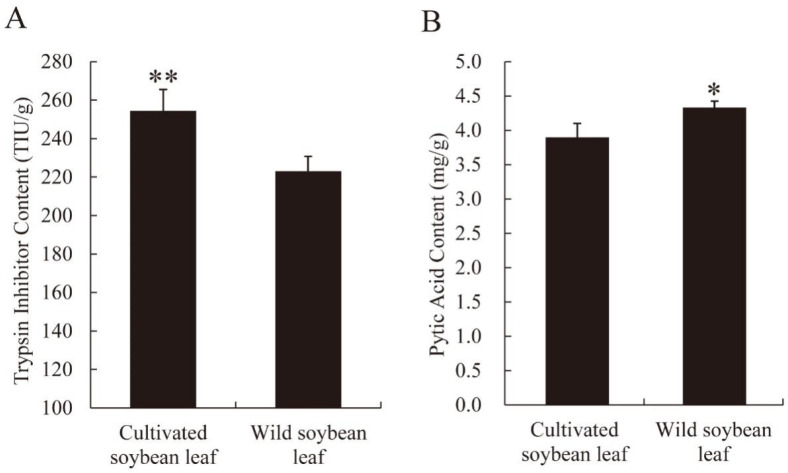
Major antinutritional factors of cultivated soybean leaves and wild soybean leaves. (**A**), trypsin inhibitor content; (**B**), phytic acid content. Asterisk denotes *p* values less than 0.05 (* *p* < 0.05, ** *p* < 0.01) by Tukey’s post hoc test.

Anti-nutritional factors can interfere with nutrient digestion and absorption in plants consumed by herbivorous insects such as *C. bilineata*. We quantified two key antinutritional factors, trypsin inhibitor and phytic acid, in leaves of both soybean types. Trypsin inhibitor levels were markedly lower in *G. soja* (wild soybean) than in *G. max* (cultivated soybean) (*p* < 0.01, Figure 1A). In contrast, phytic acid content was significantly higher in *G. soja* (wild soybean) leaves (*p* < 0.05, Figure 1B). These findings indicate that the two species differ markedly in their antinutritional profiles, which may differentially influence nutrient bioavailability when ingested by *C. bilineata*.

### 2.3. Body Weight and Nutritional Composition of Fifth-Instar Clanis bilineata tsingtauica Larvae

**Figure 2:**
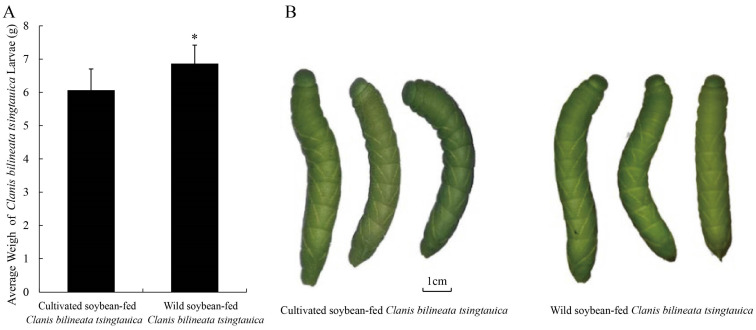
The average body weight (**A**) and morphological comparison (**B**) of *Clanis bilineata tsingtauica* larvae fed by cultivated soybean and wild soybean leaves. Asterisk denotes *p* values less than 0.05 (* *p* < 0.05) by Tukey’s post hoc test.

Dietary source significantly affected the body mass and nutritional composition of *C. bilineata* larvae. Larvae fed on *G. soja* reached a mean body mass of 6.846 g, which was 12.90% higher than that of larvae reared on *G. max* (6.066 g) (Figure 2A). Notably, larvae in the *G. soja* group exhibited a 21.10% increase in crude protein content, whereas crude fat content was 8.93% lower. Total soluble sugar content was markedly higher in the *G. soja*-fed group, accompanied by an increase in total soluble protein. In contrast, potassium content was significantly lower in larvae fed on *G. soja* (Table 2). Collectively, these results suggest that *G. soja* provides a more favorable nutritional basis for larval development, reflected in enhanced protein assimilation and carbohydrate accumulation.

### 2.4. Phyllosphere and Gut Microbiota of Fifth-Instar Clanis bilineata tsingtauica Larvae

**Figure 3:**
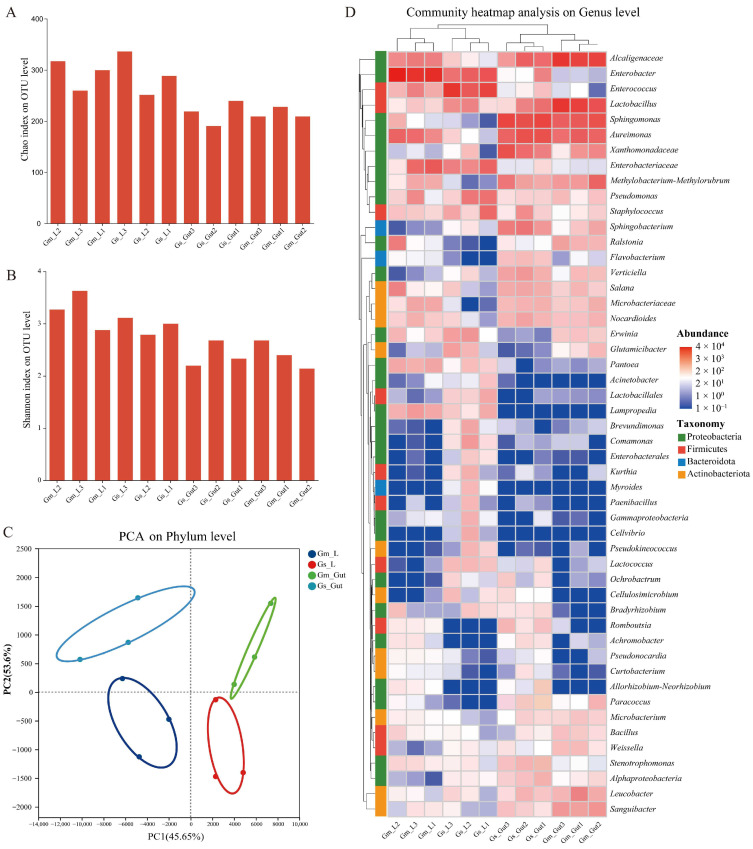
Microbial community diversity and composition in leaf and gut samples of *C. bilineata* larvae feeding on cultivated soybean (*G. max*) and wild soybean (*G. soja*). (**A**) Chao richness index; (**B**) Shannon diversity index; (**C**) PCA analysis at the phylum level and (**D**) Clustering heatmap at the genus level of phyllosphere microbiota from cultivated and wild soybean leaves, and gut microbiota of *C. bilineata* larvae fed on the respective host plants. In PCA, each point represents one biological replicate, and the ellipses indicate distribution of samples within each group. In the heatmap, red indicates higher relative abundance, whereas blue indicates lower relative abundance. Gm_L: Cultivated soybean leaves; Gs_L: Wild soybean leaves; Gm_Gut: gut microbiota of *C. bilineata* larvae fed on cultivated soybean leaves; Gs_Gut: gut microbiota of *C. bilineata* larvae fed on wild soybean leaves.

Microbial community profiling was performed on leaves of both soybean types and on the excreta of fifth-instar *C. bilineata* larvae feeding on these hosts. Across all samples, a total of 423 OTUs were identified and classified at the genus level (Supplementary Table S1). Microbial alpha diversity analysis demonstrated that both the *G. soja* and *G. max* displayed a consistent pattern where leaf samples harbored significantly higher Chao1 richness (Figure 3A) and Shannon diversity indices (Figure 3B) than gut samples, revealing substantially greater microbial species richness and overall diversity in the phyllosphere than in the intestinal microbiota of leaf-feeding insects. PCA analysis demonstrated clear separation between the gut microbial communities of larvae fed on *G. max* versus. *G. soja* and among the phyllosphere microbiota of both plant types (Figure 3C), indicating distinct community structures. Heatmap analysis of the top 50 genera further revealed distinct these differences, showing pronounced divergence in microbial composition both between the two plant phyllospheres and between the corresponding larval gut microbiota (Figure 3D). These results indicate that the two host plants recruit distinct phyllosphere microbial communities, which are reflected in the differentiated the gut microbial community composition of the *C. bilineata* larvae feeding on each host plant.

### 2.5. Comparative Analysis of Phyllosphere Microbiota of Glycine soja and Glycine max Leaves

**Figure 4:**
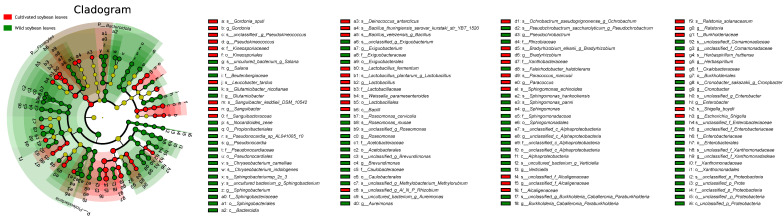
LefSe-derived cladogram illustrating differentially enriched phyllosphere microbial taxa between cultivated soybean and wild soybean leaves. Red nodes indicate taxa enriched in cultivated soybean (*G. max*), whereas green nodes indicate taxa enriched in wild soybean (*G. soja*). Yellow nodes indicate no significant difference. Concentric circles from inner to outer rings represent hierarchical taxonomic levels from phylum to species.

Linear discriminant analysis effect size (LEfSe) with an LDA threshold > 2.0 was used to identify differentially abundant phyllosphere microorganisms between *G. soja* (wild soybean) and *G. max* (cultivated soybean) leaves (Figure 4).

The analysis revealed that *G. soja* (wild soybean) leaves exhibited enrichment across a broader range of microbial taxa at multiple taxonomic levels compared with *G. max* leaves. Specifically, *G. soja* leaves were enriched in 2 phyla (*Bacteroidota*, *Proteobacteria*), 2 classes (*Alphaproteobacteria*, *Bacteroidia*), 9 orders (e.g., *Acetobacterales*, *Enterobacterales*, *Sphingomonadales*), 10 families (e.g., *Acetobacteraceae*, *Enterobacteriaceae*, *Sphingomonadaceae*), 14 genera (e.g., *Aureimonas*, *Brevundimonas*, *Sphingomonas*), and 13 species (e.g., *Chryseobacterium indologenes*, *Cronobacter sakazakii*, *Sphingomonas hankookensis*).

In contrast, *G. max* (cultivated soybean) leaves were enriched in a narrower range of taxa, including one phylum (*Firmicutes*), one class (*Bacilli*), three orders (*Burkholderiales*, *Kineosporiales*, *Lactobacillales*), seven families (e.g., *Burkholderiaceae*, *Lactobacillaceae*), nine genera (e.g., *Bradyrhizobium*, *Lactobacillus*, *Ralstonia*), and 16 species (e.g., *Bacillus velezensis*, *Lactobacillus plantarum*).

A notable enrichment of unclassified or uncultured microbial taxa was observed in the *G. soja* phyllosphere. At the class level, *G. soja* leaves harbored unclassified taxa such as *c_unclassified_p_Proteobacteria*, with this trend extending to 2 unclassified orders, 2 unclassified families, 5 unclassified genera, and 15 unclassified or uncultured species. Conversely, such unclassified taxa were scarce in *G. max* leaves, being detected only at the genus (1 taxon) and species levels (3 taxa).

### 2.6. Comparative Analysis of Gut Microbiota in Clanis bilineata tsingtauica Fed on Glycine soja and Glycine max Leaves

**Figure 5:**
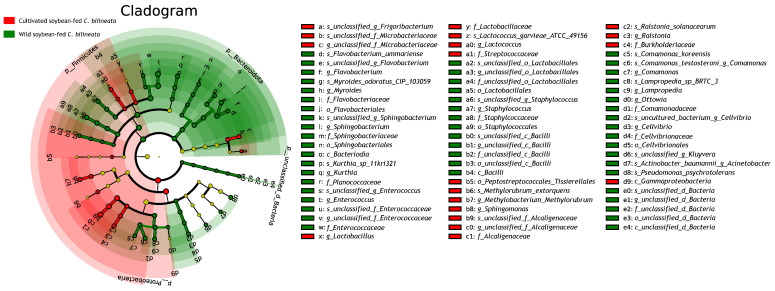
LefSe-derived cladogram illustrating differentially enriched gut microbial taxa in *C. bilineata* larvae fed on cultivated soybean or wild soybean leaves. Red nodes indicate taxa enriched in larvae fed on cultivated soybean, whereas green nodes indicate taxa enriched in larvae fed on wild soybean. Yellow nodes indicate no significant difference. Concentric circles from inner to outer rings represent hierarchical taxonomic levels from phylum to species.

LEfSe with an LDA threshold > 2.0 was performed to identify differentially abundant intestinal microbes in *C. bilineata* fed *G. soja* versus *G. max* leaves (Figure 5). The gut microbiota of larvae fed on *G. soja* leaves exhibited significant enrichment across a broader range of taxa, including 2 phyla (*Bacteroidota* and *Firmicutes*), 2 classes (*Bacilli* and *Bacteroidia*), 5 orders (*Cellvibrionales*, *Flavobacteriales*, *Lactobacillales*, *Sphingobacteriales*, and *Staphylococcales*), 7 families, 10 genera (including *Sphingobacterium*), and 8 species, several of which were also enriched in the *G. soja* phyllosphere. In contrast, the gut microbiota of larvae fed *G. max* leaves was enriched with a more limited set of taxa, including one phylum (*Proteobacteria*), one class (*Gammaproteobacteria*), one order (*Peptostreptococcales-Tissierellales*), 4 families (*Alcaligenaceae*, *Burkholderiaceae*, *Lactobacillaceae*, and *Streptococcaceae*), 5 genera (including *Lactobacillus* and *Ralstonia*), and 3 species (including *Ralstonia solanacearum*), several of which were also enriched in the *G. max* phyllosphere.

A notable enrichment of unclassified or uncultured taxa was observed in the guts of larval fed on *G. soja*, detected across all taxonomic levels, including 1 unclassified phylum (*p_unclassified_d_Bacteria*), 1 unclassified class, 2 unclassified orders, 3 unclassified families, 4 unclassified genera, and 10 unclassified or uncultured species. Conversely, the gut of larvae fed on *G. max* leaves contained substantially fewer unclassified taxa, limited to 2 genera and 3 species.

### 2.7. Potential Associations Between Gut and Phyllosphere Microbiota of Clanis bilineata tsingtauica

**Figure 6:**
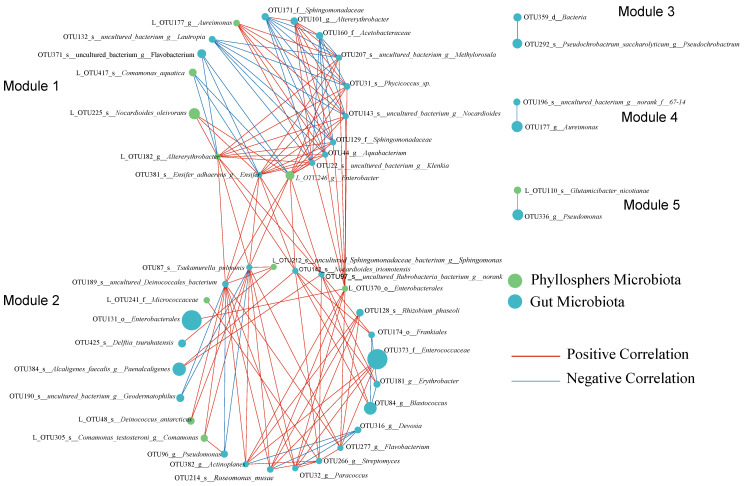
Correlation network analysis of phyllosphere microbiota and gut microbiota *C. bilineata tsingtauica* larvae. The network was partitioned into 5 modules, with Module 2 representing the dominant subnetwork. Notably, strong positive correlations between *L_OTU370_o__Enterobacterales* and *OTU131_o__Enterobacterales*, as well as between *L_OTU48_s__Deinococcus_antarcticus* and *OTU189_s__uncultured_Deinococcales_bacterium*, suggest close associations between leaf-derived and gut-associated bacterial taxa, consistent with potential diet-related microbial transfer.

Furthermore, correlation network analysis revealed distinct correlations between the gut microbiota of *C. bilineata* and the phyllosphere microbiota of the consumed leaves at the OTU level (Figure 6). The correlation network was divided into five modules: Module 1 contained 17 nodes (5 phyllosphere and 12 gut OTUs) and Module 2 containing 25 nodes (5 phyllosphere and 20 gut OTUs), while the remaining three modules each consisted of two nodes.

In contrast, negative correlations were mainly observed within Module 1 and primarily occurred between phyllosphere-phyllosphere or gut-gut OTUs, likely reflecting competitive interactions among microorganisms inhabiting the same ecological niches. Overall, correlations between phyllosphere and gut microbiota were predominantly positive, indicating potential synergistic or co-enrichment effects between leaf-associated and gut-associated microbial communities (Figure 6).

## 3. Discussion

Silkworm larvae (*Bombyx mori*) exhibit differential growth and cocoon quality depending on mulberry leaf position. Larvae fed on top leaves show higher body weight and cocoon yield due to elevated moisture and protein content [28]. Similarly, our field observations indicated that *C. bilineata* larvae preferentially fed on young upper leaves. Plant nutrients play a crucial role in insect growth and development [29]. Among these, protein serves as an important determinant of performance in phytophagous insects, which tend to prefer host plants with higher protein content to maximize nutrient acquisition under limited food intake [30]. For example, feeding on rice plants with elevated nitrogen content has been shown to enhance survival and shorten developmental duration in the brown planthopper (*Nilaparvata lugens*) [31]. In the present study, while crude protein levels did not differ significantly between the two soybean types, *G. soja* leaves exhibited significantly higher contents of crude fat, ash, neutral detergent fiber phosphorus, and calcium than *G. max* leaves (Table 1). These differences in nutrient compositions between the two host plants may contribute to the observed differences in larval performance.

Proteolytic enzyme profiles in phytophagous insects differ considerably in prevalence and catalytic efficiency among different taxa and feeding strategies [32]. Plant-derived trypsin inhibitors bind irreversibly to intestinal trypsin, forming inactive complexes that disrupt protein digestion, absorption, and utilization [33,34]. Furthermore, protease-inhibitor complexes may serve as negative feedback signals, further suppressing insect feeding behavior [35]. Collectively, these effects impair dietary protein utilization and overall food intake, ultimately hindering insect development and potentially leading to mortality due to protein deficiency. Phytic acid, a naturally occurring compound in legumes, reduces the bioavailability of essential minerals, amino acids, and proteins through chelation and complex formation [36]. Its degradation can release phosphate that becomes available to the host or gut microbiota, while phytic acid also functions as a defensive metabolite against phytophagous insects [37]. Our results showed that *G. soja* leaves contained lower trypsin inhibitor levels but higher phytic acid content compared with *G. max* leaves. In parallel, larvae fed on *G. soja* exhibited higher body weight and elevated levels of crude protein, total soluble sugar, and soluble protein. This combination of enhanced growth and nutrient accumulation likely reflects the synergistic effects of improved protein digestibility (due to reduced trypsin inhibition) and enhanced nutrient availability.

Phyllosphere-associated microbial communities play critical roles in plant productivity and ecological interactions. In this study, the phyllosphere of *G. soja* exhibited a greater microbial diversity and taxonomic breadth, harboring a more diverse and taxonomically broader community that included a substantial proportion of rare or poorly characterized taxa. In contrast, the *G. max* phyllosphere was dominated by fewer, better-characterized microorganisms. This divergence likely reflects variation in leaf-derived metabolites and volatiles that shape distinct niches for microbial colonization. Bidirectional transfer of bacteria between plant phyllospheres and insects has been documented previously [38,39,40]. Phyllosphere bacteria ingested by insects can colonize the insect guts, where they may facilitate detoxification, nutrient acquisition, and survival [41]. In larvae fed on *G. soja* leaves, the shared enrichment of several taxa between leaf and gut microbiota was consistent with possible microbial transfer, from the phylum *Bacteroidota* to the genus *Sphingobacterium*, both of which were enriched in the *G. soja* phyllosphere. A similar, albeit more limited, pattern was evident in larvae fed on *G. max* leaves. Our findings are consistent with the differences noted in phyllosphere microbiota and suggest a parallel divergence in the gut microbiota of larvae consuming these two host plants.

In addition to the direct effects of anti-nutritional factors on larval digestive physiology, growing evidence suggests that gut microbiota may play a role in mitigating these adverse effects. For instance, gut-associated bacteria of *Hermetia illucens* larvae have been shown to degrade phytic acid through the production of phytase, thereby releasing chelated minerals and improving nutrient bioavailability [37]. We speculate that the gut microbiota of *C. bilineata* may also harbor phytic acid-degrading microorganisms, which could alleviate the anti-nutritional effects through phytate degradation, although this remains to be empirically tested in future studies.

Divergence in host-associated microbial communities may contribute to the observed differences in larval nutritional profiles and growth performance. Bacteroidota primarily decomposes dietary proteins and various carbohydrates [42]. *Bacteroidota* and *Firmicutes* jointly play crucial roles in degrading cellulose and other complex carbohydrates [43]. For instance, *Prevotella* within *Bacteroidota* efficiently degrades starch and xylopolysaccharides while participating in protein metabolism [44]. These bacteria convert plant-derived proteins and non-protein nitrogen into microbial biomass for host utilization [45]. The gut microbiota of larvae fed on *G. soja* leaves showed significant enrichment in Bacteroidota and Firmicutes, which may enhance the degradation of leaf fiber and protein, thereby increasing nutrient assimilation and supporting larval growth. In contrast, the less diverse gut microbiota associated with *G. max* feeding may result in less efficient nutrient extraction, potentially contributing to the reduced growth performance of larvae on this host plant.

Collectively, our results suggest that the superior growth performance of larvae fed on *G. soja* can be attributed to a combination of three interconnected factors: (1) a more favorable profile of antinutritional factors (lower trypsin inhibitors) that enhances protein digestibility; (2) a richer and more diverse phyllosphere microbial community that serves as an inoculum for gut microbiota; and (3) the establishment of a gut microbial community enriched in fibrolytic and proteolytic taxa that promotes more efficient nutrient utilization from host plant material. These mechanistic insights have practical implications for *C. bilineata* farming. This study positions *G. soja* as a viable alternative feed source for *C. bilineata*, offering the dual benefits of reducing competition with grain soybean production and advancing the sustainability of insect rearing systems. In the broader context of insect farming, the silkworm (*Bombyx mori*) represents a successful model for large-scale rearing, benefiting from centuries of domestication, well-established artificial diets [46]. *C. bilineata* remains still at an early stage of domestication, with current rearing systems largely dependent on fresh host plant material rather than formulated artificial diets. A limitation of this study is that the proposed microbial transfer remains correlative rather than causal. Future studies should use microbiota transplantation or probiotic supplementation to establish causality and develop microbiota-based strategies for industrial *C. bilineata* rearing.

## 4. Materials and Methods

### 4.1. Plant Material

The cultivated soybean (*G. max*) variety used in this study was Qihuang 34, while the wild soybean (*G. soja*) consisted of homozygous lines originally collected from Dongying and maintained in the laboratory [47]. The experiment was initiated in early June 2024 at the Yellow River Delta Modern Agriculture Research Institute, Shandong Academy of Agricultural Sciences, Shandong, China. Both soybean types were grown under standard field management in plots of approximately 50 m^2^, using 0.6 m row spacing and 0.15 m intra-row spacing. Each plot was covered with insect-proof nets one week prior to the release of *C. bilineata tsingtauica* eggs (Supplementary Figure S1A,B).

### 4.2. Inoculation of Clanis bilineata tsingtauica Larvae

Forty days after sowing when plants had developed sufficient leaf biomass, commercially purchased *C. bilineata* eggs were attached to the abaxial surface of *G. max* and *G. soja* leaves. One egg cluster (30–50 eggs) was placed per m^2^, to allow natural hatching (Supplementary Figure S1C,D).

### 4.3. Plant Material Sampling

Larval development of *C. bilineata* larvae in each plot was monitored regularly. When approximately 70% of larvae reached the fifth instar, fully expanded upper young leaves from *G. max* and *G. soja* were collected. Field observations indicated that *C. bilineata* predominantly feeds on young upper leaves; therefore, these leaves were used for nutritional component analysis and phyllosphere microbial sequencing. For both *G. max* and *G. soja* plots, samples were collected from three locations with high larval density. Immediately after sampling, leaves were flash-frozen in liquid nitrogen and stored at −80 °C for subsequent analyses.

### 4.4. C. bilineata Larvae and Feces Sampling

Sampling of *C. bilineata* larvae and their feces began three days after leaf collection. Fifth-instar larvae were collected from high-density areas in both *G. max* and *G. soja* plots. At each of three independent sampling sites per plot, 6–10 larvae were collected. Larvae were immediately flash-frozen in liquid nitrogen and stored at −80 °C. Fresh feces were collected from the same locations, flash-frozen in liquid nitrogen and stored at −80 °C for further experiments.

### 4.5. Determination of Leaf and Larval Indicators

The determination of leaf nutritional components (crude fat, crude protein, crude fiber, ash, neutral detergent fiber, acid detergent fiber, phosphorus (P), and calcium (Ca)) and larval nutritional indicators (crude protein, crude fat, soluble sugar, soluble protein, and potassium (K)) followed the procedures described by Li et al. (2011) [48]. Phytic acid and trypsin inhibitor contents were determined following Liu et al. (2020) [49], with minor modifications. In brief, 5 g of leaf tissue was extracted with 40 mL of sodium sulfate-hydrochloric acid solution on a shaker for 4 h. The mixture was centrifuged at 8000 rpm for 5 min, and the supernatant was adjusted to 50 mL with the same solution and then filtered. For phytic acid purification, 2 mL of extract was mixed with 2 mL of 15% trichloroacetic acid (TCA) solution and incubated at 4 °C for 2 h. After centrifugation, 2 mL of the supernatant was adjusted to pH 6.0–6.5 with 1 mol/L NaOH and diluted to 30 mL with distilled water. Standard curve preparation and quantification followed national standard procedures (GB5009.153-2016) [50]. For trypsin inhibitor activity, 1 g of leaf tissue was extracted with 50 mL of 0.01 mol/L NaOH solution, adjusted to pH 9.5 ± 0.1 using 0.1 mol/L hydrochloric acid (HCl) solution, and incubated at 4 °C for 24 h. The extract was equilibrated at 25 °C, diluted to 100 mL with water, and analyzed using the national standard method (GB5009.224-2016) [51]. For each analytical parameter, measurements were performed in triplicate.

### 4.6. Microbial Sequencing and Bioinformatic Analyses

Total microbial genomic DNA was extracted from leaf and fecal samples using *EasyPure*^®^ Genomic DNA Kit (EE101-01) (Transgen, Beijing, China). The V5-V6 region of the bacterial 16S rRNA gene was amplified with primers 799F (5′-AACMGGATTAGATACCCKG-3′) and 1193R (5′-ACGTCATCCCCACCTTCC-3′) [52]. Amplified PCR products were pooled in equimolar amounts, and DNA libraries were constructed using the SMRTbell prep kit 3.0 (Pacific Biosciences, Menlo Park, CA, USA). Sequencing was performed on the PacBio Sequel IIe System (Pacific Biosciences, Menlo Park, CA, USA) by Majorbio Bio-Pharm Technology Co., Ltd. (Shanghai, China). High-fidelity (HiFi) reads were generated using circular consensus sequencing via SMRT Link v11.0. Optimized HiFi reads were clustered into operational taxonomic units (OTUs) using UPARSE 7.1 [53,54] at a 97% sequence similarity threshold. The most abundant sequence in each OTU was selected as the representative sequence, and chloroplast sequences were removed during manual filtering. Taxonomic classification of each representative sequence was performed using the RDP Classifier version 2.2 [55] with a confidence threshold of 0.7. Putative metagenome functions were predicted using PICRUSt2 (2.6.0) [56] based on representative OTU sequences. Bioinformatic analyses of phyllosphere and gut microbiomes were carried out using the Majorbio Cloud platform (https://cloud.majorbio.com) [57].

### 4.7. Data Analysis

All data were subjected to one-way analysis of variance (ANOVA). Mean differences were compared using Tukey’s post hoc test, and differences were considered significant at *p* < 0.05 and *p* < 0.01. Statistical analyses were performed using SPSS v 17.0 (SPSS Inc., Chicago, IL, USA).

## 5. Conclusions

This study evaluated the effects of nutrients, anti-nutritional factors, and phyllosphere microorganisms in wild (*G. soja*) and cultivated (*G. max*) soybean plants on larval nutrition and gut microbiota composition in *C. bilineata*. Compared with *G. max*, *G. soja* showed higher levels of several nutritional components, including crude fat, ash, neutral detergent fiber, calcium, and phosphorus, as well as lower levels of plant trypsin inhibitors. Larvae fed on *G. soja* leaves exhibited higher biomass, protein content, and sugar content than those fed on *G. max* leaves. *G. soja* leaves exhibited a stronger capacity for microbial recruitment, harboring greater microbial diversity and abundance. Gut microbiota analysis revealed that, consistent with the differences in phyllosphere microorganisms between *G. soja* and *G. max*, larvae fed on the two leaf types also exhibited distinct gut microbial communities. Based on these results, *G. soja* leaves may serve, to some extent, as a partial substitute to *G. max* for *C. bilineata* rearing. By utilizing *G. soja* as a feedstock, the competition with cultivated soybean production can be alleviated, while also enabling the exploitation of saline-alkali lands where *G. soja* is naturally adapted. Furthermore, the distinct phyllosphere and gut microbial profiles associated with *G. soja* feeding provide a basis for the development of microbiota-targeted strategies, such as probiotic supplementation, to improve larval performance in future artificial diet formulations. These results provide a scientific basis for the further development of *C. bilineata* production systems.

## Figures and Tables

**Table 1:** Nutritional components comparison of cultivated soybean and wild soybean leaves.

Indicators	Cultivated Soybean Leaf	Wild Soybean Leaf
Crude fat (%)	2.17 ± 0.19	5.61 ± 0.29 **
Crude protein (g/kg)	242.51 ± 0.73	240.73 ± 1.35
Crude fiber (%)	9.68 ± 0.29 **	7.30 ± 0.27
Ash content (%)	9.62 ± 0.07	11.07 ± 0.04 **
Neutral detergent fiber (%)	21.00 ± 0.38	23.75 ± 0.39 **
Acid detergent fiber (%)	13.21 ± 2.78	14.24 ± 1.80
P (g/kg)	2.38 ± 0.05	2.52 ± 0.02 *
Ca (g/kg)	3.41 ± 0.03	4.05 ± 0.02 **

Note: Asterisk denotes *p* values less than 0.05 (* *p* < 0.05, ** *p* < 0.01) by Tukey’s post hoc test. Data represent mean ± SE.

**Table 2:** The comparison of nutritional components on the fifth-instar of *Clanis bilineata tsingtauica* fed by cultivated soybean and wild soybean.

Indicators	*G. max*-Fed *C. bilineata*	*G. soja*-Fed *C. bilineata*
Crude protein (g/kg)	555.02 ± 3.16	672.14 ± 3.88 **
Crude fat content (g/kg)	205.82 ± 4.62 *	187.44 ± 2.75
Total soluble sugar (mg/g)	8.96 ± 0.36	21.27 ± 0.45 **
Total soluble protein (mg/g)	24.71 ± 0.65	26.35 ± 0.49 *
K (g/kg)	2.44 ± 0.01 **	2.26 ± 0.01

Note: Asterisk denotes *p* values less than 0.05 (* *p* < 0.05, ** *p* < 0.01) by Tukey’s post hoc test. Data represent mean ± SE.

## Data Availability

The original contributions presented in this study are included in the article and Supplementary Materials. Further inquiries can be directed to the corresponding authors.

## Supplementary Materials

The following supporting information can be downloaded at: https://www.mdpi.com/article/10.3390/ijms27083442/s1.
