# Supplementary material for: Comparative Effects of Glycine max and Glycine soja Leaves on Clanis bilineata tsingtauica Rearing Performance
Source: Int J Mol Sci. 2026 Apr 11;27(8):3442. doi: 10.3390/ijms27083442 (PMC13117024; doi:10.3390/ijms27083442)
Supplement: Supplementary file 1 [file ijms-27-03442-s001.zip › Supplementary Figure S1.pdf]

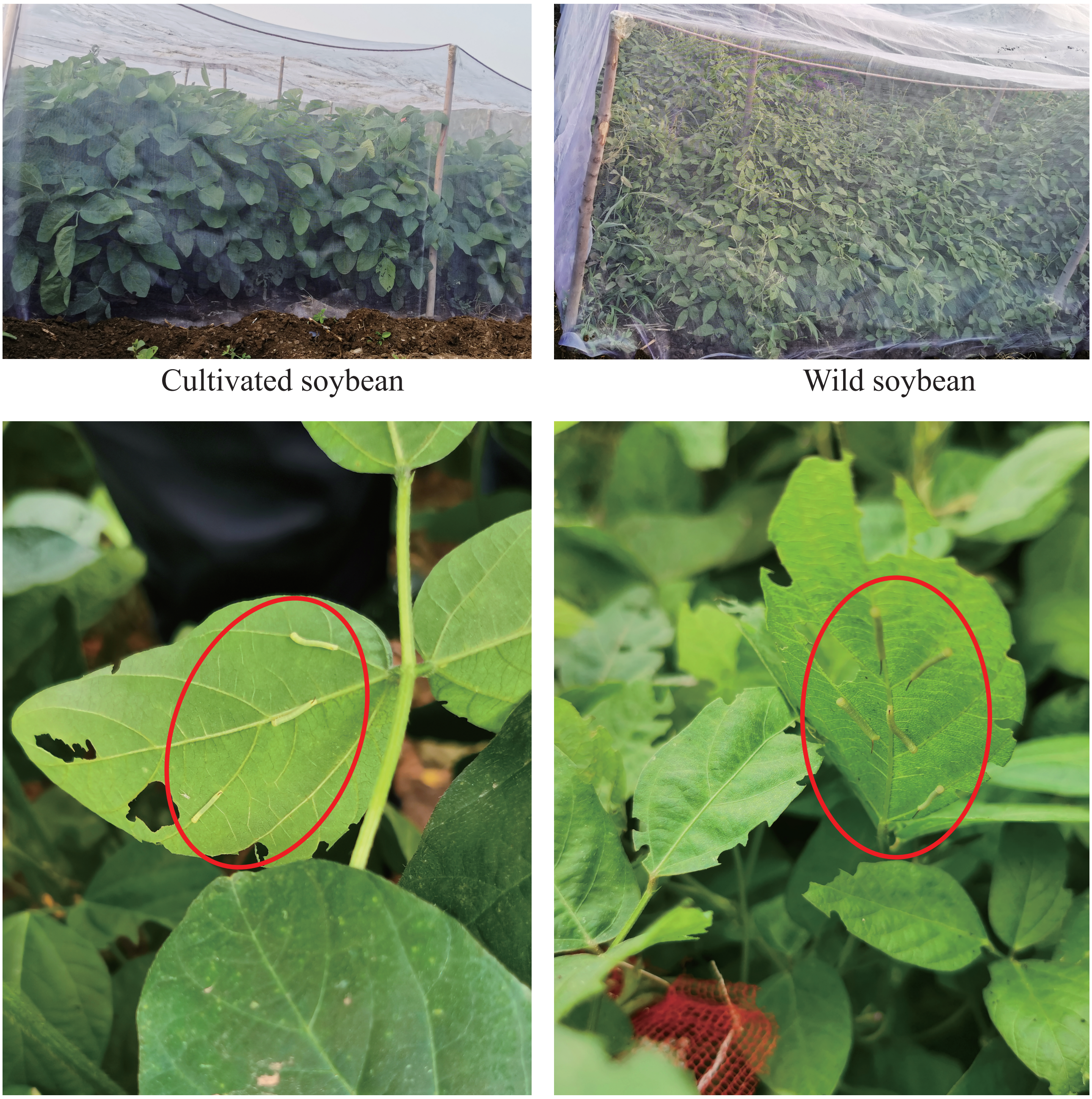

A
Cultivated soybean

B
Wild soybean

C
Cultivated soybean-fed *C. bilineata*

D
Wild soybean-fed *C. bilineata*

Supplementary Figure S1: Cultivation of cultivated soybean and wild soybean, and the incubation of *C. bilineata* larvae. (A) Cultivated soybean, (B) Wild soybean, (C) Cultivated soybean-fed *C. bilineata*, (D) Wild soybean-fed *C. bilineata*.
